# Scalable approach towards specific and ultrasensitive cation sensing under harsh environmental conditions by engineering the analyte–transducer interface†

**DOI:** 10.1039/d0na01042a

**Published:** 2021-05-17

**Authors:** Sudeshna Mondal, Chandramouli Subramaniam

**Affiliations:** Department of Chemistry, Indian Institute of Technology Bombay Powai 400076 Mumbai India csubbu@chem.iitb.ac.in

## Abstract

Affordable and high-performing sensing platforms are becoming increasingly critical for sustainable environmental monitoring and medical diagnostics. Such miniaturized and point-of-care sensing platforms need to overcome the fundamental tradeoff between ultrahigh sensitivity and specificity while retaining the dynamic concentration range and robustness of operation. Therefore, designing scalable and robust sensors poses an escalating and immediate demand in a rapidly automated society. Addressing this demand, we demonstrate a cable-type electrochemical sensing platform exhibiting rapid (10 s), extremely reliable (RSD <5%) and ultrahigh sensitivity (ppb levels) towards K^+^, Cd^2+^ and Hg^2+^ found in complex biofluids such as human perspiration and effluent water. The sensor delivers quantifiable performance even with 10 μL of analyte without any requirement of purification or preconcentration and thereby overcomes an important bottleneck for on-field diagnostics. The backbone of the sensor consists of single-walled carbon nanotubes (CNTs) that are conformally coated on affordable cellulose yarns to form ideally non-Faradaic, electrically conductive, capacitive electrodes (CNT-thread). Subsequent coaxial coating of such CNT-threads with an appropriate ionophore membrane (IM) realizes the working electrode exhibiting uniformity in the surface coverage of the ionophore leading to reliable and directly quantifiable signals. Furthermore, we show that the extensive CNT-thread–IM interface is critical to achieve ultrahigh sensitivity and robust operability. Importantly, the design approach adopted is universal and scalable for a range of cations such as K^+^, Hg^2+^ and Cd^2+^. Thus, the sensor delivers ultrasensitive detection of K^+^ from very low volumes (10 μL) of human perspiration that contains a wide range of other ions (Cu^2+^, Zn^2+^, Cd^2+^, Fe^2+^, NO_3_^−^, Cl^−^) at 1000-fold higher ionic strength along with bioinorganic suspended matter (dead cells, organelles). This eliminates any sample treatment or preconcentration requirements thereby overcoming a major obstacle for point-of-care applications. Furthermore, both multicomponent and multivariate analyses are demonstrated with the sensing device targeting portable and wearable applications.

## Abstract 2

Ultra-selective quantification of K^+^, Cd^2+^ and Hg^2+^ in perspiration and effluent waste-water at sub-ppm levels is demonstrated using real-time analytes in media with high ionic strength, suspended bio-particulates and 3-fold higher concentration of chemical interferents.

## Graphical abstract

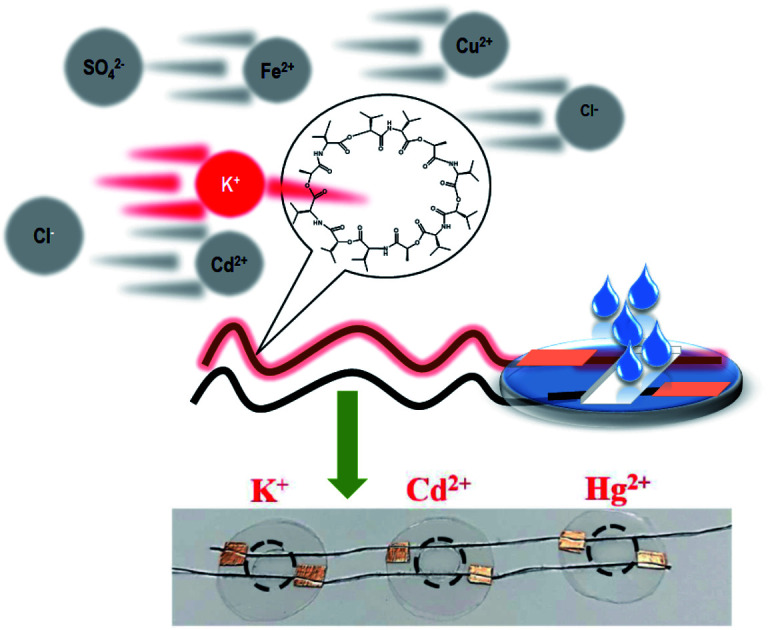

## Introduction

The thrust for sustainable development through the internet-of-things (IoT) has created a huge demand for portable, efficient and reliable sensors in the form of point-of-care medical diagnostics and water quality monitoring.^1^ In fact, access to clean energy and water forms primary objectives of the United Nations Sustainable Development Program and relies heavily on achieving efficient and affordable water quality monitoring systems.^2^ Such sensors need to be deployed in a variety of applications, that differ widely in operational conditions, such as healthcare, food products, pharmaceuticals, security, industrial safety and environmental and agricultural monitoring. In this context, portable, lab-on-chip electrochemical sensors^3–5^ with a rapid detection protocol and ease of operation are in demand. Furthermore, high specificity and ultrasensitivity are mutually exclusive tradeoffs in the domain of sensors. An ideal sensing platform aims to overcome several limitations encountered in conventional modes of analysis such as extensive pretreatment and sample collection, access to trained personnel and cumbersome equipment. Portable chemical sensors which are wearable and point-of-care capable of non-invasively monitoring electrolytes and metabolites in various biofluids, such as sweat,^6^ tears,^7^ and saliva^8^ cater to the expanding internet of things.^9,10^ The versatility of such platforms would extend to the assessment of soil nutrition^11,12^ and environmental monitoring leading to scientific and precision agriculture.^13–16^

Conventional detection platforms like atomic absorption spectroscopy (AAS),^17^ inductively coupled plasma optical emission spectrometry (ICP-AEOS)^18^ and inductively coupled plasma mass spectrometry (ICP-MS) are standard techniques for metal ion detection, these being highly sensitive and reliable;^19,20^ however, they require sophisticated instrumentation and are thus not cost-effective or easily accessible. Hence, electrochemical platforms which are point-of-care are of immediate demand for detection of these ions.^21,22^ Furthermore, the wide concentration range of operation has been demonstrated with multi-modal techniques such as chronoamperometry and differential pulse voltammetry ensuring amenability for AI-based detection protocols.^23–25^

A major challenge in realizing such sensors is their reliability and reproducibility of operation, particularly under widely varying real-time, on-field conditions. This is mainly controlled by the uniformity in surface coverage of the receptors within (a) a sensor and (b) across multiple batches of sensors. Furthermore, ultrahigh molecular-level sensitivity has always been associated with unreliable and irreproducible responses from the sensor.^26^ This predominantly originates from non-specific interactions between the receptor and analyte at the transducing interface. Consequently, monitoring of a single analyte across a wide range of concentration becomes extremely challenging and yet, highly desirable.^11^

This study focusses on three different analytes that are relevant and important from the context of both medical diagnostics and environmental monitoring. In this context, potassium ions are a major constituent of perspiration that controls several physiological parameters. Present in an optimum concentration of 195.5–250 ppm, deficiency of potassium causes high blood pressure, polyurea, muscle paralysis, breathing problems, irregular heart rhythms, and constipation.^27–29^ Additionally, quantitative sampling of perspiration poses several challenges due to the (a) low sampling volumes (microlitres), (b) large amount of biofouling agents acting as blockers and (c) presence of chemically similar metabolites like glucose and lactose. Similarly, heavy metal ions such as cadmium and mercury are major contaminants of fresh water sources and pose several health hazards due to their carcinogenic, mutagenic and accumulative nature in ecosystems. High levels of cadmium exposure through water can lead to respiratory disorders, and liver as well as kidney problems in humans.^30–32^ Mercury poisoning through exposure to high levels of mercury has given rise to several diseases like Minamata disease, and Hunter–Russell syndrome which has further established the toxic and dangerous effects of mercury in water.^33–35^ Thus, the design and development of an affordable and scalable sensing platform to reliably detect such important analytes in harsh environments has been an enduring unmet challenge.

Addressing these challenges, we present a scalable approach to engineer the transducer–analyte interface and thereby achieve reliable, ultrasensitive detection of a wide variety of cations present in versatile analytes in harsh environments, relating to physiological and water-quality monitoring. The sensor exhibits identical performance under standardized laboratory conditions as well as with complex real-time samples such as body fluids (sweat) and turbid lake water. Both these applications involve very specific detection of K^+^, Cd^2+^ and Hg^2+^ in ppb levels in the presence of a large excess (1000 times) of potential interferents such as Na^+^, Cu^2+^, Zn^2+^, Cd^2+^, NO_3_^−^, Cl^−^ and particulate matter that can block and deactivate the sensing surface. The method demonstrated a potassium ion sensor capable of detecting its losses from perspiration at point-of care, *i.e.* while performing exercise or physical activity, with high sensitivity (2.15 mA ppm^−1^) and signal reliability (RSD ≤3%) operating over a linear range of 10–500 ppm with a limit of detection (LOD) as low as 30 ppm.

The cation sensing platform utilizes CNTs in the form of a conductive CNT-thread for effective transduction of analyte–receptor interactions. The chemical receptor is homogeneously coated onto to the CNT-thread in the form of a polymeric membrane such that the receptor/analyte signal is recorded in the form of an electrochemical signal following standard techniques of cyclic voltammetry (CV), chronoamperometry (CA) and differential pulse voltammetry (DPV). The novelty of the work extends to its ability to detect multiple cations in a similar transducer/receptor interface wherein the receptor changes according to the metal ion it is specific to, *e.g.* valinomycin is used as a receptor for detection of K^+^ ions, whereas *N*,*N*,*N*′,*N*′-tetrabutyl-3,6-dioxaoctanedi(thioamide) and *N*,*N*′-dibenzyl-4,13-diaza-18-crown-6 are used as chemical receptors for Cd^2+^ and Hg^2+^ offering a linear range of 0.5–2.5 ppb, signal reliability (RSD ≤5.5%) and a limit of detection (LOD) of 0.12 ppb for the sensing platform. This produces a versatile electrochemical platform wherein the electrochemical specificity is not only governed by chemical specificity but also by the extent of binding of these metal ions onto their respective receptors, which in turn is used to enhance parameters like the specificity, sensitivity and linear range of these sensors.

## Experimental section

Super-growth single-walled carbon nanotubes (CNTs, length: 500 μm, diameter: 3 nm) were synthesized using water-assisted chemical vapour deposition.^36^ The potassium ionophore (valinomycin) >98% pure, cadmium ionophore (*N*,*N*,*N*′,*N*′-tetrabutyl-3,6-dioxaoctanedi(thioamide)) (NTDT), mercury ionophore (*N*,*N*′-dibenzyl-4,13-diaza-18-crown-6) (NDDC6) and high molecular weight poly(vinyl chloride) (PVC), tetrahydrofuran, 2-nitrophenyl octyl ether (NPOE, >99%), sodium tetraphenyl borate (NaTPB, >99%), and analytical grade salts KCl, Cd(NO_3_)_2_, HgCl_2_, Zn(NO_3_)_2_, Ca(NO_3_)_2_, Fe(NO_3_)_2_, Mg(NO_3_)_2_ and sodium deoxycholate (NaDOC) were purchased from Sigma Aldrich and used without further purification. 100 ppm standard solution (AAS) grade Cd and Hg were diluted and used for the electrochemical sensing studies. Acetate and phosphate buffers were used to regulate the pH of the medium. Ultrapure Milli-Q water was used throughout the study.

### Preparation of the transducer: CNT-thread

High aspect ratio (∼10^5^) CNTs were dispersed through probe sonication (50% power at 26 °C using a PKS-750FM, PCI Analytics) using NaDOC as an anionic surfactant^37^ to produce aqueous conductive dispersions (CNT inks). Commercially available multiple cellulose yarns (diameter = 150 μm) were dip-coated through the CNT-ink to immobilize the CNTs onto the cellulose thread and thereby form electrically conductive, flexible substrates. This was consecutively air-dried, washed in ethanol and redried in air to form a CNT-thread which acted as a transducer.

### Preparation of the receptor: ionophore membrane

The ionophore was solubilized in a homogeneous medium using PVC, plasticizer NPOE and anion-excluder NaTPB (30 : 5 : 55 : 10) dissolved in 5 mL of tetrahydrofuran. This resulted in cocktails which were highly stable. The CNT-thread was dip-coated into the ionophore cocktail for immobilization of the ionophore onto the CNT-thread. The CNT-thread was then air dried, resulting in the formation of a uniform coating of the ionophore membrane (IM) onto it after the evaporation of THF. Thus, the receptor in the form of a membrane was directly coated onto the CNT-thread, hence acting as the working electrode for sensing.

## Material characterization

The morphology of the CNT-thread and ionophore coated CNT-thread was studied using Field-Emission Scanning Electron Microscopy (FE-SEM, Zeiss ultra 55 FESEM, 5 kV). X-ray photoelectron spectroscopy was conducted on an AXIS Supra (Al Kα 1486.6 eV). Confocal Raman spectroscopy was performed with a WiTeC MicroRaman using a 532 nm Nd-YAG excitation laser source (5 mW power). Confocal spectral mapping of the samples was carried out at diffraction-limited spatial resolution by mounting the samples on a piezo controlled XYZ translational stage. Each spectral image corresponds to 10 000 spectra.

### Device fabrication

The IM coated thread acted as the working electrode (WE) and the bare CNT-thread acted as a reference–counter electrode (RE). The WE and RE were then each attached to conductive Cu tape and act as input and output terminals. The distances between the WE and RE and the sampling area were both fixed using PET as the packaging material. A constant distance of separation between the two electrodes was maintained using circular disks of PET (radius = 23 mm) as the packaging material. A circular opening (radius = 5 mm) in one of the PET sheets provided the sampling window while maintaining complete flexibility. A number of such sensors were directly integrated on the same CNT-thread by using IM coated in different parts of the same WE. It is to be noted that such a device assembly process does not require any additional current collector.

### Electrochemical measurements

All electrochemical measurements were conducted using a Biologic SP-300 potentiostat in a two-electrode configuration with the IM coated CNT-thread acting as the WE and the pristine CNT-thread acting as the RE. All experiments in the laboratory were performed after purging the sample with pure N_2_ gas (99.99% purity) for 5 minutes and the results collected after iR_drop_ corrections.

### Real-time sampling and analysis

Real-time testing of various metal ions like K^+^, Cd^2+^ and Hg^2+^ in a wide range of samples was used for testing the applicability of the universal cation testing platform. Real-time sweat samples from a healthy volunteer were collected during 1 hour of vigorous exercise at 15 minute intervals. The K^+^ content from the sensor was then compared with the actual value of K^+^ content in sweat obtained through ICP-AES. For the testing of Cd^2+^ and Hg^2+^ from water, samples were collected from Powai Lake in Mumbai, India. These lake water samples were tested for their Cd^2+^ and Hg^2+^ content and the recovery percentages were calculated after comparison with those obtained through standard ICP-AES techniques.

## Results and discussion

The performance of a chemical sensor is largely dependent on the specificity of the receptor and the effectiveness of the transducing element. While the former dictates the selectivity of the sensor, the latter controls its sensitivity and reliability. Therefore, it is important for both these to work in unison for achieving ultrahigh sensitivity without compromising on reliability and specificity. Thereby, the interface between the transducer and the receptor becomes an important design element that determines the performance of the sensor. The transducing element should possess (a) high charge carrier concentration for rapid transduction of the receptor/analyte interaction, (b) high specific surface area guaranteeing high sensitivity of transduction, and (c) chemical inertness to avoid any interference during detection. Based on these parameters, we employ single-walled carbon nanotubes (CNTs) as the transducing element in the electrochemical platform.

In spite of these advantages, the major challenge for utilizing CNTs is their uncontrolled aggregation driven by van der Waals forces. This often leads to severe deterioration in the active surface area, hindering the performance and reliability of the sensor. We circumvent this challenge by achieving a highly uniform and stable dispersion of CNTs in an aqueous medium by employing NaDOC as an anionic surfactant. NaDOC stabilizes the CNTs through electrostatic surface interactions and enables uniform coating of the CNTs over any hydrophilic surface. Furthermore, the conformal coating can be realized over a variety of two-dimensional and three-dimensional surfaces. The surface loading of the CNTs on the cellulose yarn was gravimetrically estimated to be 0.125 mg cm^−1^, thus ∼10 mL of CNT-ink would generate 75 cm of CNT-thread (50 devices).^37^ Thereby, the CNTs were coated on three-dimensionally porous hydrophilic cellulose yarn (CNT-thread). Such a CNT-thread exhibits ohmic electrical conductivity (25 ± 0.5 S cm^−1^) with negligible variation over length scales that are at least three orders of magnitude longer than individual CNTs. A microscopic examination of such a CNT-thread reveals the formation of an interpenetrating and continuous network of CNTs with p-type, ohmic electrical conductivity. The surfactant is subsequently removed by washing with ethanol. This enhances the inter-CNT contacts and also avoids any interference from the surfactant during the working of the sensor. Besides contributing to 10^15^ times enhancement in electrical conductivity, the CNT-thread presents the synergistic combination of an electrochemically inert functional electrode with high specific surface area (800 m^2^ g^−1^).^11^ Additional advantages of employing such CNT-threads for devices are the complete elimination of any current collector and mechanical flexibility, in comparison to other reports that compromise the performance and mechanical flexibility due to the presence of the current collector.

**Fig. 1:**
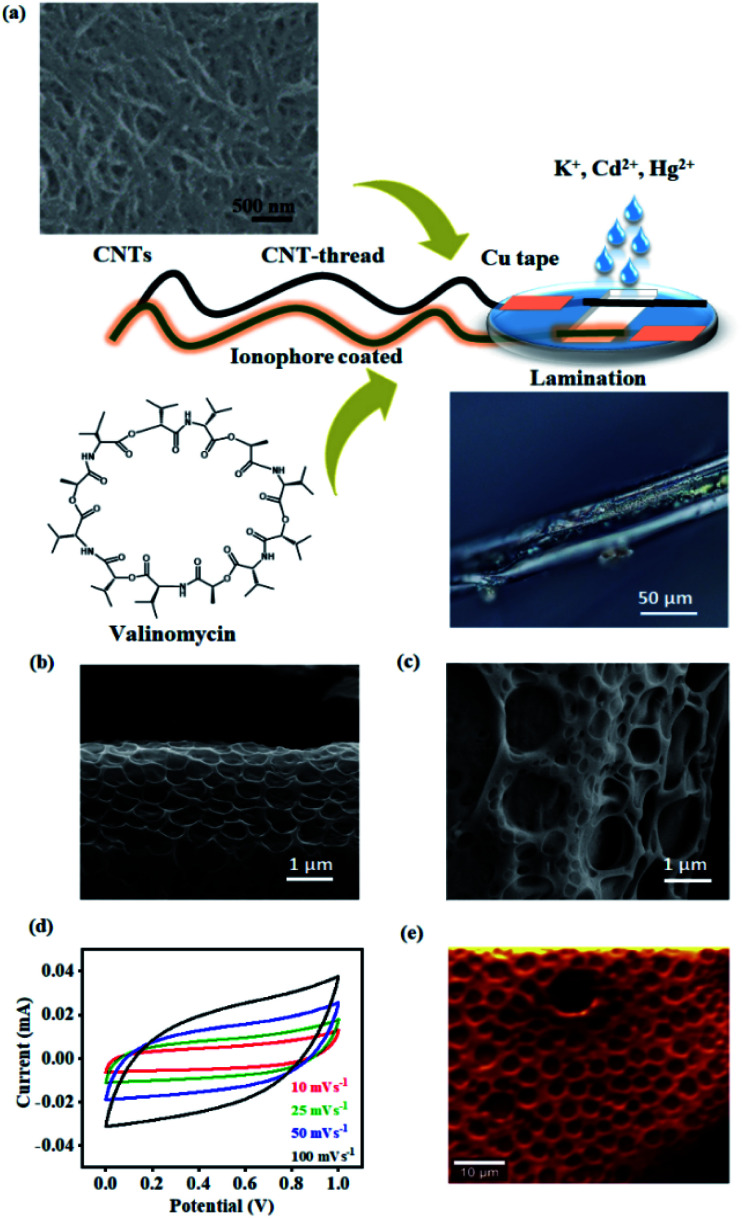
(a) Schematic of the steps involved in the fabrication of the sensor such as coating of CNTs and the ionophore (valinomycin for K^+^), followed by lamination to realize the final electrochemical sensor. Appropriate ionophores (NTDT for Cd^2+^ and NDDC6 for Hg^2+^) were employed for other analytes. The 3D optical microscopy image of the ionophore coated CNT-thread and (b)–(c) SEM of the CNT-thread are provided. (d) Graph demonstrating the ideal polarizability and capacitive nature of the CNT-thread. (e) Raman spectral mapping of the ionophore membrane based on the *ν*_bending_ (N–H) at 1505 cm^−1^.

The second major challenge deals with achieving uniform surface coverage of the receptor (ionophore) on the transducer, which directly controls the reliability and sensitivity of the sensor. Conventional approaches to establish an effective interface between the receptor and transducer include self-assembly, covalent anchoring and electrostatic layer-by-layer assembly.^38–40^ Such approaches often result in uncontrollable and non-uniform surface coverage of the receptor leading to variation in the number density of receptors. This has a direct bearing on the signal response and raises concerns in both quantification and reliability of the device. We overcome this challenge by uniformly dispersing the ionophore in a polymeric matrix. The resulting blend is conformally coated onto the CNT-thread and ensures uniformity in the surface distribution of the ionophore. Subsequently, the CNT-thread is dip-coated through the blend resulting in a coaxial, three-dimensional encapsulation of the transducer by the sensing layer (polymer ionophore membrane). Thus, the design principle demonstrated is effective in overcoming the major shortcomings of conventional approaches while ensuring scalability of the device fabrication. Furthermore, the electrochemical inertness and the voltage stability of the pristine CNT-thread make it ideal as the RE of the sensor. The final sensing device thus consists of the pristine CNT-thread acting as the reference electrode and the ionophore membrane coated CNT-thread serving as the working electrode. Finally, the device is packaged between two PET sheets that facilitate (a) constant distance between the reference and the working electrode, (b) facile contact with copper adhesive tapes and (c) a uniform sampling window for improved signal reliability and reproducibility (Fig. 1(a)).

Specificity and sensitivity of detection are two critical and mutually exclusive parameters that determine the effectiveness of a sensor. The fabrication approach permits the enhancement of specificity in two major stages. The first stage of specificity arises due to the chemical affinity between the ionophore and analyte. However, such chemical specificity of interaction alone is insufficient for achieving high specificity. Accordingly, the second stage of specificity is affected by monitoring the strength of interaction between the receptor and the analyte as probed through electrochemical techniques, *i.e.* by monitoring the surface energies at the electrode/electrolyte interface, thereby the specificity of interaction is derived by monitoring the strength which is specific to a particular analyte and receptor. Given that real-time detection exclusively involves sensing of one analyte in an ocean of multiple other chemically similar analytes, such a two-pronged approach ensures higher specificity and lower selectivity coefficient during real-time detection.

Accordingly, the K^+^ sensor comprises the valinomycin ionophore membrane that is uniformly and coaxially coated on the three-dimensional CNT-thread. This technique also enables precise control over the thickness of the ionophore membrane (1.6 μm) and thereby achieves a balance between the ionophore–analyte and ionophore–transducer interfaces. The universality of the design principle allows similar sensors to be fabricated for a range of other cations such as Cd^2+^ and Hg^2+^ using appropriate ionophores. This approach also enables facile incorporation of an anion excluder (NPOE) which is important to minimize the interference from the counter ions and thereby helps in achieving a low selectivity coefficient (*vide infra*).

The porous structure of the ionophore membrane and the uniformity of its coverage around the CNT-thread is confirmed from the SEM images (Fig. 1(b) and (c)). The chemical inertness of the CNT-thread and its ability to behave as an ideally polarized electrode was demonstrated through cyclic voltammograms recorded in acetate buffer (pH 4.5) with two pristine identical CNT-threads acting as the electrodes (Fig. 1(d)). The resulting CVs exhibit ideal capacitive behaviour with symmetric cathodic and anodic segments. Importantly, the absence of any Faradaic component and the linear dependence of current on the scan rate confirm the absence of any parasitic side reactions (Fig. S1†). The specific capacitance estimated (15.6 F g^−1^ at 100 mV s^−1^) confirms the formation of an extensive interfacial electrical double layer.

Furthermore, we note that ohmic behaviour is completely retained after such polymeric sheathing indicating facile charge transduction through the CNT-thread. The complete infiltration of this polymeric matrix into the micro and mesopores of the CNT-thread creates an extensive and continuous interface between the ionophore membrane and the CNT-thread. Raman spectral mapping of the ionophore membrane reveals vibrational modes at 627 cm^−1^ and 1505 cm^−1^ corresponding to *ν*_stretching_(N–C=O) and *ν*_bending_(N–H) thereby establishing the effectiveness of the coating (Fig. 1(e)).

**Fig. 2:**
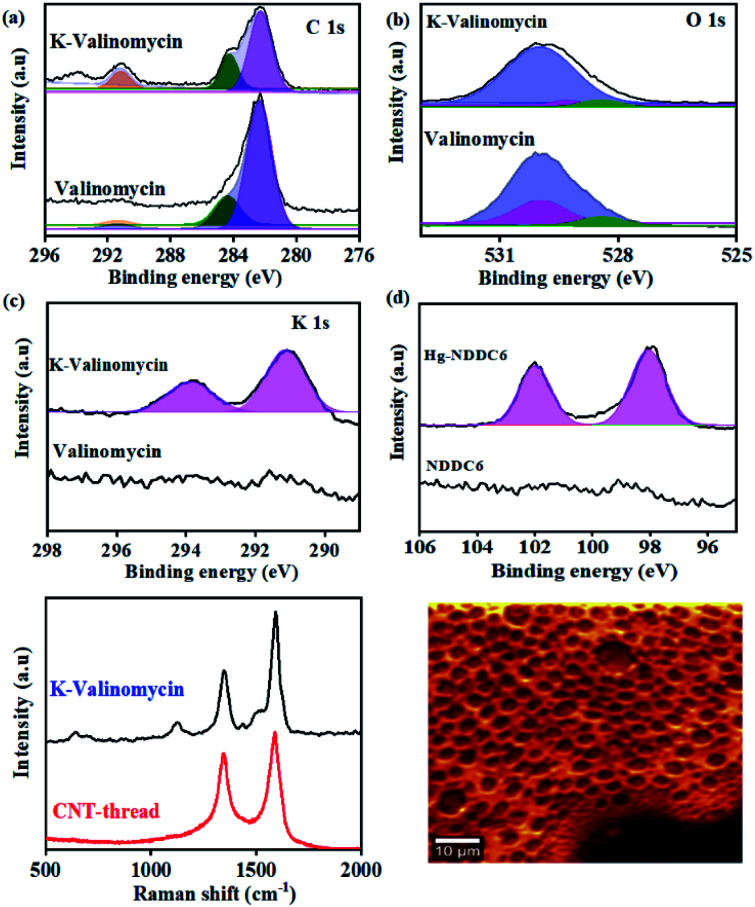
XPS spectra of (a) C 1s, (b) O 1s and (c) K 2p in the valinomycin ionophore membrane before and after addition of 10 ppm of K^+^. (d) XPS spectra of Hg 4f in the ionophore membrane before and after addition of 10 ppm of Hg^2+^ on the NDDC6 polymer membrane. (e) Raman spectra of CNT-thread and K-valinomycin membrane coated CNT-thread. (f) Raman spectral mapping of the ionophore membrane based on the *ν*_stretching_ (C–C) at 1100 cm^−1^.

The chemical affinity between the metal ion (analyte) and its corresponding ionophore was monitored using surface sensitive X-ray photoelectron spectroscopy. Pure valinomycin exhibits the characteristic C 1s peak corresponding to sp^2^ C=C at 284.6 eV, along with broad peaks at 284.4 eV and 289 eV that are attributed to C–N and C=O (Fig. 2(a)). Subsequent to the binding of K^+^, the O1s peak observed at 529.9 eV in pure valinomycin is downshifted to 529.3 eV indicating binding of K^+^ through the O–C=O groups (Fig. 2(b)). Further confirmation arises from the appearance of K1s peaks at 291 eV and 293 eV originating from the spin–orbit coupled 2p_1/2_ and 2p_3/2_, which is completely absent in the case of pure valinomycin (Fig. 2(c)). Similarly, distinct peaks originating from Hg 4f_5/2_ and 4f_7/2_ and Cd 3d_3/2_ and 3d _5/2_ are observed in the case of Hg–N binding and Cd–S binding after exposure of the ionophore membranes to 10 ppm of Hg and Cd, respectively (Fig. 2(d), S2†). The consistent peak shifts observed after binding of the cation to the ionophore confirms the charge transfer-based interactions.

The uniformity in the spatial distribution of valinomycin is investigated through micro-Raman spectral mapping (Fig. 2(e) and (f)). The Raman spectrum of the CNT-thread exhibits the characteristic D-band and G-band originating from the presence of CNTs. Additional vibrational modes at 1500 cm^−1^ were observed upon encapsulation of the CNT-thread with the valinomycin ionophore membrane. These modes originate from the aliphatic C=C and C–C that make up the ionophore membrane. Importantly, the 1124 cm^−1^ vibrational mode corresponding to the O–C=O exhibits a red shift upon exposure to K^+^, in agreement with the charge transfer based interactions in XPS. The spectral map generated, using the intensity of the 1100 cm^−1^ peak, across an area of 2500 μm^2^ exhibits uniformity in both spatial distribution of K^+^ and its concentration profile (Fig. 2(f), S3†).

**Fig. 3:**
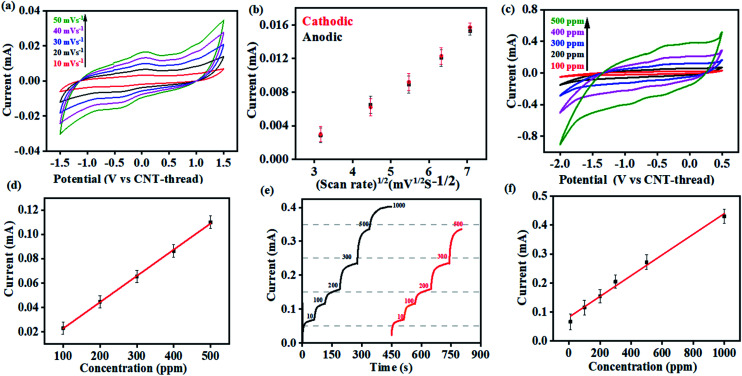
(a) Cyclic voltammograms recorded with 10 ppm of K^+^ at different scan rates in acetate buffer (pH 4.5) using the sensing device. (b) Randles–Sevick plot derived from (a) indicating the diffusion-based kinetics of the K^+^/K redox couple at the electrode/electrolyte interface. (c) Cyclic voltammograms for the sensor at different concentrations of K^+^ ranging from 100 ppm to 500 ppm (pH 4.5, 25 mV s^−1^). (d) Corresponding calibration plot of current *vs.* the concentration of K^+^. (e) Chronoamperometric response of the sensor in acetate buffer (pH 4.5) at different concentrations of K^+^. Response from the same device is shown in black and red to demonstrate the reusability of the device. (f) Corresponding calibration plot derived from (e).

Cyclic voltammograms of the K^+^ sensor in 10 ppm KCl electrolyte (0.2 M acetate buffer at pH 4.5) exhibit distinct redox peaks corresponding to the K^+^/K redox couple in both the cathodic (−0.4 V) and the anodic (0.04 V) sweeps (Fig. 3(a)). The Faradaic charge-transfer based interaction between the K^+^ in the electrolyte and the ionophore coated CNT-thread is evident from experiments conducted at varying scan rates (10 mV s^−1^ to 50 mV s^−1^, Fig. 3(a)). The Randles–Sevick fit derived from the CVs indicates a linear dependence of both the cathodic and anodic currents on the square root of scan rates and confirms one electron transfer. The identical slopes also confirm the reversibility of the reaction and indicate the diffusion-based kinetics of the electrochemical process (Fig. 3(b)). Furthermore, cyclic voltammograms were recorded over a wide range of K^+^ concentration (100 ppm to 500 ppm, 25 mV s^−1^) and exhibit excellent linearity in response with a sensitivity of 2.15 mA ppm^−1^ (Fig. 3(c) and (d)).

The large change in current with respect to the concentration of the analyte and the linearity of response are both important and challenging aspects to realize in an electrochemical sensing platform. Importantly, the device exhibits negligible interference from the presence of Na^+^, Cu^2+^, Zn^2+^, Cd^2+^, NO_3_^−^, Cl^−^, and CH_3_COO^−^ at concentrations that are at least three orders of magnitude (1000 ppm) higher than the analyte (K^+^) concentration. Besides establishing the efficiency of this fibre-based sensing platform, the ease of fabrication also augurs well for its practical applicability.

**Fig. 4:**
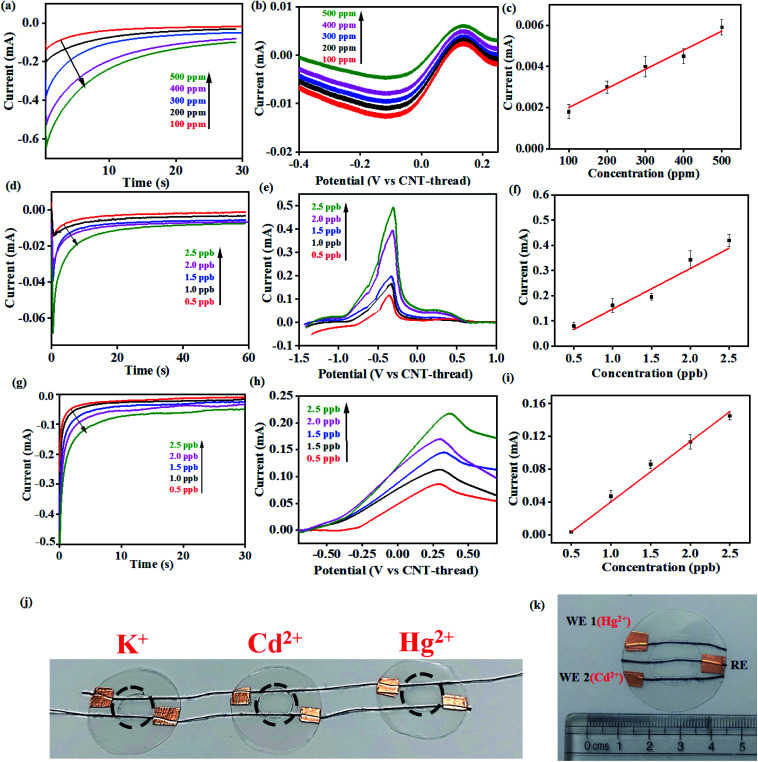
(a) Chronoamperometric curves and (b) differential pulse voltammograms recorded with different concentrations of K^+^ (100–500 ppm). (c) Corresponding calibration plots including relative standard error (each measurement was repeated 5 times) for 100–500 ppm K^+^ in acetate buffer (pH 4.5). (d) Chronoamperometric and (e) differential pulse voltammograms (deposition potential: −1.2 V) for Cd^2+^ at concentrations ranging from 0.5 ppb to 2.5 ppb. (f) Corresponding calibration plots including relative standard error (each measurement was repeated 5 times) for 0.5–2.5 ppb Cd^2+^ in acetate buffer (pH 4.5). (g) Chronoamperometric curves and (h) differential pulse voltammograms (deposition potential: −1.2 V) of Hg^2+^ at concentrations ranging from 0.5 ppb to 2.5 ppb and (i) corresponding calibration plots including relative standard error (each measurement was repeated 5 times). (j) Photograph of the multi-parametric sensing platform which can simultaneously detect three different metal ions with minimum interference. (k) Multiplexing capability with the same sensing device being used for detection of both Hg^2+^ (WE 1) and Cd^2+^ (WE 2) with the same sample.

Multi-modal sensing offers the advantage of involving data analysis and principal component analysis for e-nose^41,42^ based applications. Accordingly, the response of this sensor was evaluated through multiple electrochemical techniques enabling (a) multi-modal analysis and (b) cross-correlation between the responses obtained from different techniques. It is important to note that no change in the design or working of the device was required for any of the electroanalytical techniques demonstrated. Accordingly, chronoamperometric *i*–*t* curves were recorded at constant voltage (−0.4 V) to evaluate the electrochemical performance of the sensor (Fig. 3(e)). Sequential increase in the concentration of K^+^ was affected while simultaneously monitoring the chronoamperometric current. As is the case with CV, chronoamperometry also exhibits a strong linear correlation between the concentration of K^+^ and the resulting current. The LOD estimated was 30 ppm calculated using:1
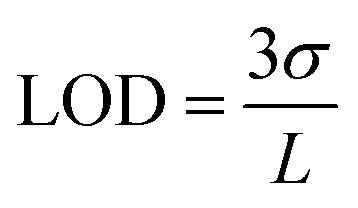
where *σ* is the standard deviation of the blank solution, and *L* denotes the slope of the calibration curve.^43^ The LOD further confirms the reliability of the sensing device over a wide concentration range spanning three orders of magnitude (10–1000 ppm). The CA and the DPV studies performed on the K^+^ sensor using 100–500 ppm concentrations of the analyte also exhibit linearity in response (Fig. 4(a)–(c)).

This electrochemical platform, demonstrating excellent operational range, response time and limit of detection comparable to other reports (Table S1†), has been extended to other cationic heavy metal ions such as Cd^2+^ and Hg^2+^. This involved a simple replacement of valinomycin (ionophore for K^+^) with NTDT and NDDC6 for Cd^2+^ and Hg^2+^ respectively. The rise time leading to saturation of signal for both Cd^2+^ and Hg^2+^ was <10 s (Fig. 4(d) and (g)). The anodic segment of DPV provided currents that are linearly dependent on the concentration of the analyte with sensitivities of 0.078 mA ppb^−1^ (Cd^2+^) and 0.073 mA ppb^−1^ (Hg^2+^) (Fig. 4(e)–(f) and Fig. 4(h)–(i)). This further establishes the usage of the platform for real-time detection because of the significant advantage of low LOD and operational range of working as compared to other reports (Table S1†) for both Cd^2+^ and Hg^2+^ ions from water.

The resulting devices exhibit similar superlative sensing for the respective analytes. Furthermore, the use of the mechanically flexible and robust CNT-thread as the electrochemical platform provides the opportunity to combine multiple sensors on the same platform and thereby achieve multianalyte, quantitative tracking. Such an approach for design and fabrication leads to an origami-inspired foldable array of electrochemical sensors (Fig. 4(j)). Furthermore, each of the sensors in the array can be operated either individually or simultaneously using a variety of electrochemical techniques. Identical experiments were carried out for Cd^2+^ and Hg^2+^ using devices that are tethered together using common electrodes that contain spatially isolated ionophore domains. Importantly, the operation of one device does not hinder the other devices establishing their operability in both a standalone manner and in a multiplexing array. Such an array provides a distinct advantage over other ISE assemblies^44–46^ that can operate only in a stand-alone fashion. Further versatility of the sensor is demonstrated by operating the CNT-thread as a common RE while utilising two different WEs for Hg^2+^ and Cd^2+^ within the same sensor (Fig. 4(k)). The WE in such a device consists of appropriate ion-selective membrane coated CNT-threads (NTDT for Cd^2+^ and NDDC6 for Hg^2+^). The readout collected with individual sets of RE–WE shows identical responses with the sensor shown in Fig. 1(a) and 4(j). Thus, a unique multiplexing functionality arising out of the configurational advantage and the pliability of the electrodes is achieved. This also enables tailoring the sensor as per the on-field requirements by direct mix–match of various WEs and REs.

The real-time functioning of the sensor depends on two important factors: (a) the capture of the specific ion by the ionophore and (b) the charge-transfer interaction between the analyte and the ionophore, which is finally picked up by the transducing channel (CNT-thread) and converted into a readable electrical signal as captured by the potentiostat. The pπ–d interactions between the ionophore and the specific metal ion indicate the chemical nature of the origin of the sensitivity of the sensor while the high sensitivity arises from the highly conductive CNT-thread. Another important aspect of the sensor is its selectivity which is one of the main criteria for its usage for on-field real-time applications in sweat monitoring (K^+^) as well as environmental monitoring (Cd^2+^ and Hg^2+^).

In order to study the effect of pH on the sensor performance, chronoamperometric tests were carried out with analytes (K^+^) under acidic (pH 3.0 and pH 4.5) and near-neutral conditions (pH 7.0). The valinomycin-functionalized ionophore membrane exhibits the highest sensitivity (0.95 mA ppm^−1^) under near-neutral conditions across the range of K^+^ concentrations (100–500 ppm) tested (Fig. S4(a)†). The chronoamperometric response of K^+^ exhibits a strong dependence on the pH of the medium, with the saturating current being the highest under neutral conditions. This is attributed to the competitive and non-selective interaction of the valinomycin cavity with solvated H^+^. Accordingly, the saturating current for K^+^ is found to decrease with decrease in pH and concurrent increase in the concentration of H^+^.

**Fig. 5:**
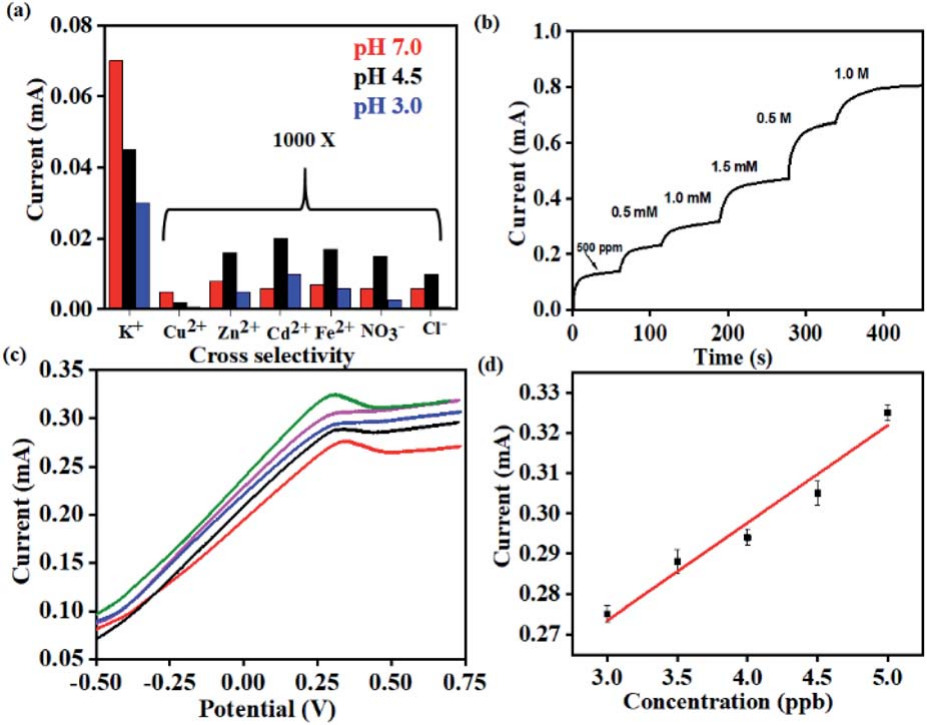
(a) Selectivity studies on the K^+^ sensor in the presence of 1000 times excess concentrations of counter ions and potential interferents recorded at different pH. (b) Real-time studies on the K^+^ sensor using human sweat samples (10 μL) after spiking with 500 ppm of 1.0 M K^+^. (c) Differential pulse voltammetric studies on real-time lake water samples with spiked concentrations of Hg^2+^. (d) Corresponding calibration plots recording the current with spiked concentrations of Hg^2+^.

Importantly, the selectivity of the sensor was evaluated by measuring similar chronoamperometric curves in the presence of other potential interfering ions (Cu^2+^, Zn^2+^, Fe^2+^, Cd^2+^ and anions such as Cl^−^ and NO_3_^−^) at ∼1000 times higher concentrations than K^+^ at different pH (Fig. 5(a)). When the K^+^ concentration is 1 ppm, the concentrations of interfering ions (Cu^2+^, Zn^2+^, Fe^2+^, Cd^2+^, Cl^−^ and NO_3_^−^) were 1000 ppm. Identical devices were used to elucidate the response for each of these ions through independent measurements. All such devices exhibit the highest current-response for K^+^ while the lowest saturating current was obtained for the interfering ions (Cu^2+^, Zn^2+^, Fe^2+^, Cd^2+^, Cl^−^ and NO_3_^−^) (Fig. S4(b–d)†). This behavior was uniformly observed across all the three pH conditions tested. Accordingly, we establish the pH dependent sensing behavior of the proposed electrochemical platform.

Thus, the sensor is highly selective for K^+^ in spite of the presence of other potential interferents in a large excess (1000 times). The selectivity coefficient, calculated using the chronoamperometric plot, ranges from 8.90 × 10^−3^ for Cd^2+^ to 4.49 × 10^−3^ for Cl^−^. The selectivity coefficient was estimated using the following equation:2
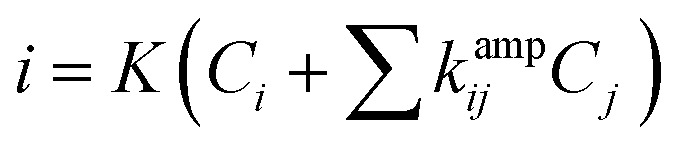
where *C*_*i*_ and *C*_*j*_ are concentrations of the target analyte (*i*) and interfering ion (*j*), respectively and *k*^amp^_*ij*_ is the amperometric selectivity coefficient.^47^ Similar ranges of selectivity coefficients were obtained for Cd^2+^ and Hg^2+^ in the presence of an excess (1000 ppb) of other ions such as Fe^2+^, Zn^2+^, Cu^2+^, K^+^, NO_3_^−^, Cl^−^, and SO_4_^2−^. This establishes the robustness and the specificity of the sensors, an aspect critical for real-time applications (Fig. S5†). The observed selectivity of the sensor originates from the combination of (a) chemical affinity and (b) strength of interaction between the receptor and the analyte. The sensor is designed to capture the strength of interaction between the receptor and analyte. This results in enhanced performance of the sensor, particularly in harsh and complex environments. This also forms an important aspect of the work and presents an alternative and effective strategy, compared to conventional electrochemical sensing that aims to capture the chemical affinity between the ionophore and analyte.

Consequently, the sensors were utilized for real-time analysis to address practical challenges in healthcare monitoring and environmental pollution. Real-time sweat samples were collected from a healthy volunteer over a period of 1 hour while performing aerobics. 20 uL of the collected sweat sample was spiked with increasing concentrations of K^+^ and its chronoamperometric response was recorded (Fig. 5(b)).

No additional sample treatment or filtration was carried out. The collected sweat sample gave a saturation current of 0.1 mA within <10 s corresponding to K^+^ saturation of 500 ppm. This was in excellent agreement with the values provided through ICP-AES. Subsequent analysis was carried out with the same sensor by artificially spiking the K^+^ concentration of the collected sweat sample. The sensor exhibits characteristically distinct current outputs that exhibited a strong dependence on the concentration of K^+^. This is consistent with the earlier observations and also establishes the specificity of the device. The study was extended for real-time detection of Hg^2+^ in water samples drawn from a nearby lake (Powai, Mumbai). The Hg^2+^ sensor exhibits ultrahigh sensitivity with the detection limit going down to 3 ppb (Fig. 5(c)). The consistent reduction peaks at 0.37 V matched with that of the signals in Fig. 4 (h) from which it was evident that the sensor could accurately sense Hg^2+^ from real-time samples without any interference from other ions which are present in real-time samples. The inset shows the linearity in current response with increase in the spiked concentration of Hg^2+^ and the real-time sensitivity was calculated to be 0.080 mA ppb^−1^. Hence, the platform demonstrates real-time applicability with robust performance.

## Conclusions

In summary, a universal design principle for qualitative and quantitative detection of cations (K^+^, Hg^2+^, Cd^2+^) of environmental and diagnostic significance is demonstrated. The CNT-thread based electrodes exhibit bifunctional behaviour as both the counter and reference electrodes, besides being an accurate charge-electron transducer. The approach of coaxially encompassing such a CNT-thread with a polymeric blend containing the appropriate receptors provides excellent versatility to detect a wide range of cations. Detailed studies shown here for K^+^, Cd^2+^ and Hg^2+^ exhibit the unique combination of a very low LOD, linearity across a wide concentration range and ultrahigh selectivity. Such coaxially assembled electrodes offer extremely uniform surface coverage of receptor molecules leading to high reliability of sensing and present a unique approach that overcomes reliability issues with other conventional routes. Furthermore, the multiplexing through a variety of electrochemical DC techniques provides the opportunity for further incorporation of data analysis-based algorithms. This can be extended for future development of similar platforms for a plethora of redox active species across wide ranging environmental conditions.

## Conflicts of interest

There are no conflicts to declare.

## Supplementary Material

NA-003-D0NA01042A-s001
