# Supplementary material for: Scalable approach towards specific and ultrasensitive cation sensing under harsh environmental conditions by engineering the analyte–transducer interface
Source: Nanoscale Adv. 2021 May 17;3(13):3752–61. doi: 10.1039/d0na01042a (PMC9418407; doi:10.1039/d0na01042a)
Supplement: NA-003-D0NA01042A-s001 [file NA-003-D0NA01042A-s001.pdf]

Electronic Supplementary Material (ESI) for Nanoscale Advances.
This journal is © The Royal Society of Chemistry 2021

# Scalable approach towards specific and ultrasensitive cation sensing in harsh environmental conditions by engineering the analyte-transducer interface

Sudeshna Mondal,[a] Chandramouli Subramaniam[a]

[a] Department of Chemistry, Indian Institute of Technology Bombay, Powai, Mumbai 400076, India.

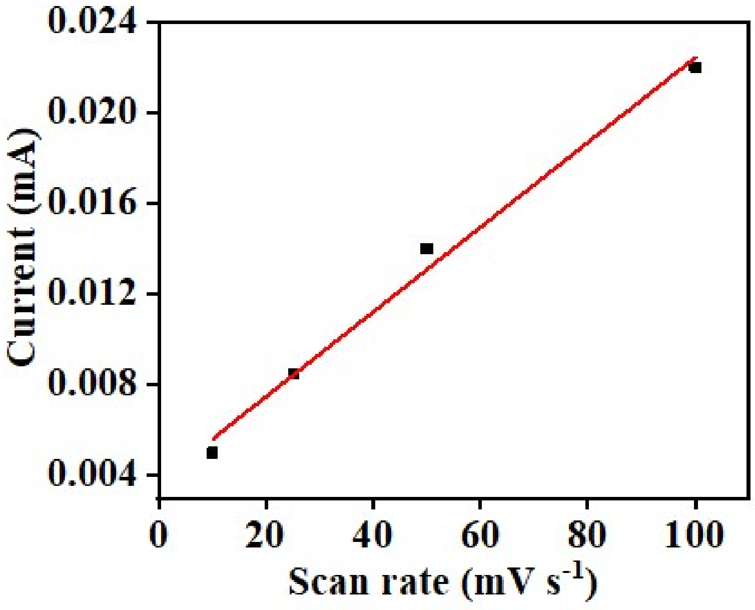


**Figure S1. Randles Sevick plot showing characteristics of an ideally polarized electrode with CNT-thread.**

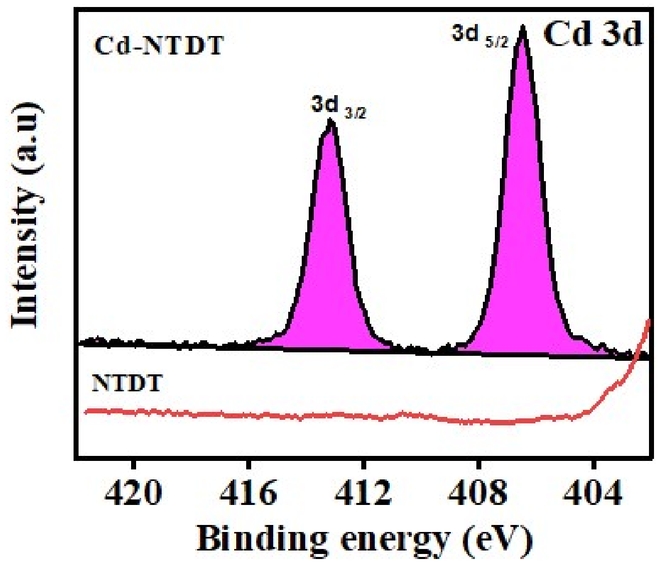


**Figure S2. XPS spectra of Cd 3d in Cd-NTDT before and after addition of 10 ppm of $Cd^{2+}$.**

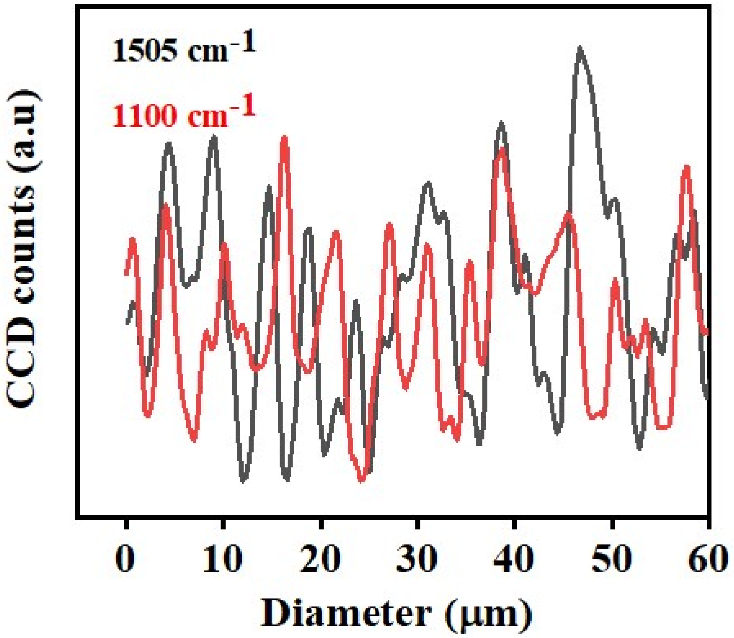


**Figure S3. Line intensity profile of $\nu_{bending}$ (N-H) at 1505 $cm^{-1}$ and $\nu_{stretching}$ (C-C) at 1100 $cm^{-1}$ of K-Valinomycin ionophore membrane as obtained from Raman spectral mapping.**

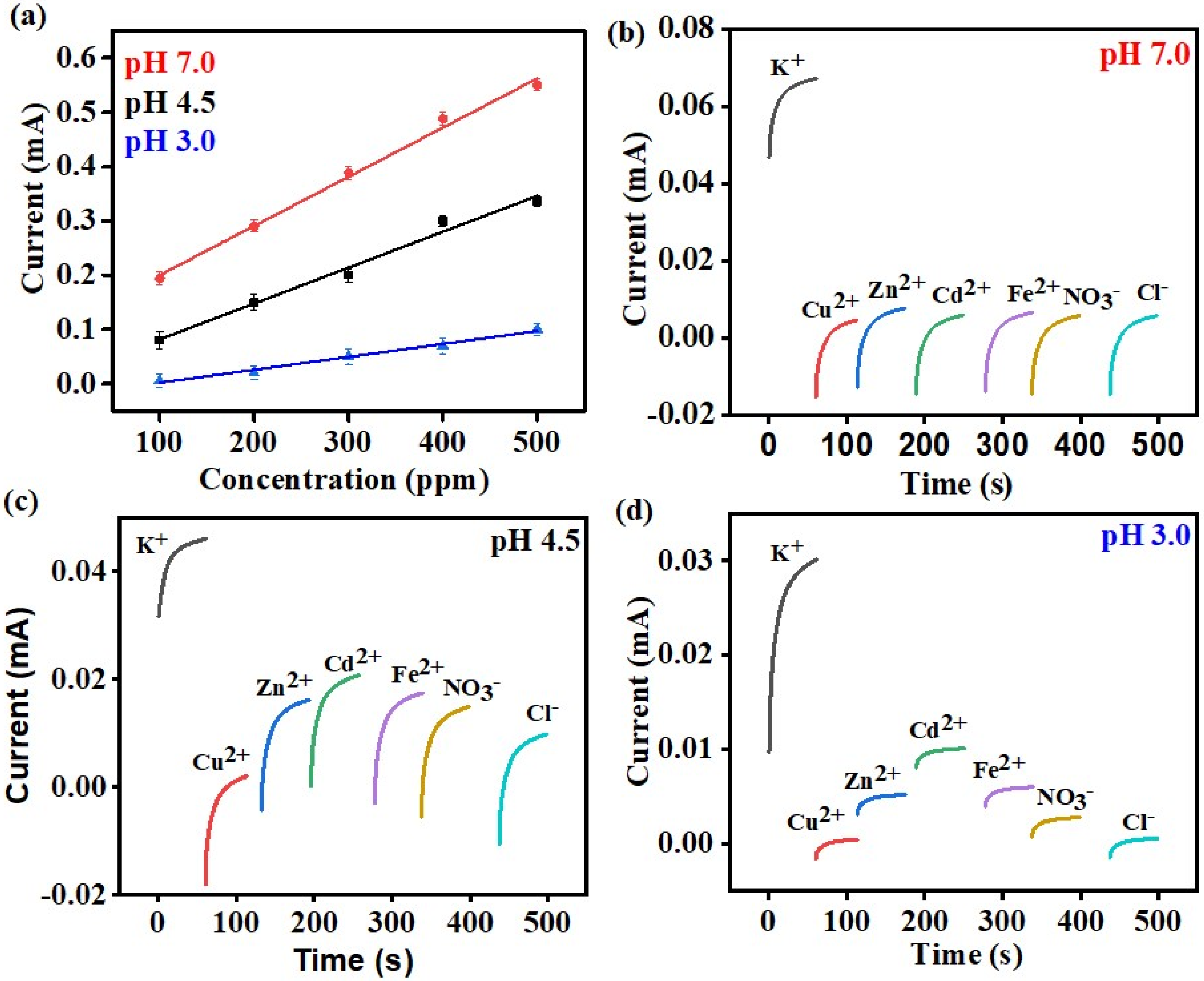


**Figure S4. (a) Chronoamperometric studies of $K^+$ at different pH and concentrations ranging from 100 ppm-500 ppm. Selectivity studies on the $K^+$ sensor at (b) pH 7.0, (c) pH 4.5 and (c) pH 3.0.**

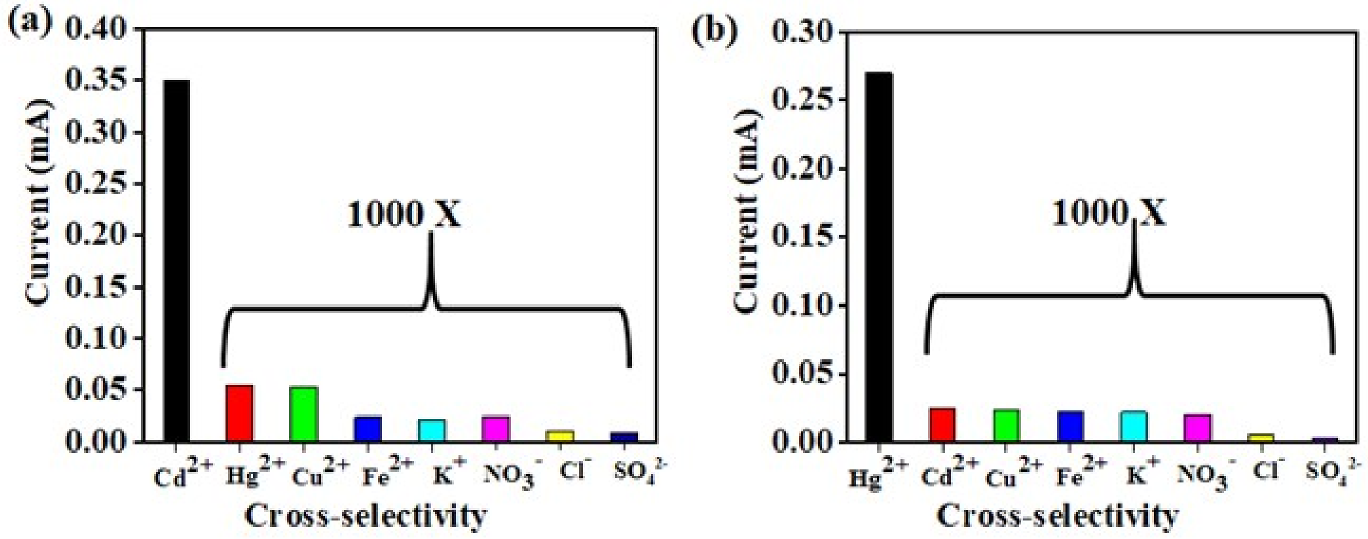


**Figure S5. (a) Selectivity studies on the $Cd^{2+}$ sensor in presence of 1000 times excess concentrations of counter ions. (b) Selectivity studies on the $Hg^{2+}$ sensor in presence of 1000 times excess concentrations of counter ions.**

**Table S1. Analytical characteristics of point-of-care electrochemical platforms for detection of $K^{+}$, $Cd^{2+}$ and $Hg^{2+}$**

| **Electrode ($K^{+}$)** | **Type of transduction** | **Linear range (ppm)** | **Limit of detection (ppm)** | **Response time (s)** | **Reference** |
|---|---|---|---|---|---|
| Textile/polyurethane/ CNTs | solid-contact | $10- 10^4$ | 1 | 5 | 1 |
| CNTs/ cotton | solid-contact | $10-10^4$ | 10 | 60 | 2 |
| Carbon/PEDOT/PET | solid-contact | $10-10^4$ | 0.01 | - | 3 |
| Adhesive carbon tape | Inner solution | $100-10^4$ | 0.039 | 5 | 4 |
| **Valinomycin- IM** | **solid contact** | **10 – 500** | **30** | **10** | **Present work** |
| **Electrode ($Cd^{2+}$)** | **Type of transduction** | **Linear range (ppb)** | **Limit of detection (ppb)** | **Response time (s)** | **Reference** |
| Au/Ti-RGO | solid contact | 1.0-120 | 1.0 | - | 5 |
| Paper strip/SWNTs/ P3OT | solid contact | - | 0.13 | - | 6 |
| RGO/CNT/Bi | solid contact | 20 -200 | 0.6 | - | 7 |
| **NTDT-IM** | **solid contact** | **0.1 -2.5** | **0.12** | **10** | **Present work** |
| **Electrode ($Hg^{2+}$)** | **Type of transduction** | **Linear range (ppb)** | **Limit of detection (ppb)** | **Response time (s)** | **Reference** |
| CNT-thread | solid contact | 300-1200 | 0.2 | 120 | 8 |
| EDTAPANI/MWCNTs/SPCE | solid contact | 0-2.0 | 0.003 | - | 9 |
| Au/PET | solid contact | 0-300 | - | 120 | 10 |
| **NDDC6-IM** | **solid contact** | **0.1-2.5** | **0.10** | **10** | **Present work** |

## References


1. M. Parrilla, R. Cánovas, I. Jeerapan, F. J. Andrade, J. Wang, *Adv Healthc Mater.* 2016, 5(9), 996-1001.
2. T. Guinovart, M. Parrilla, A. G Crespo, F. X Rius, F. J. Andrade, *Analyst* 2013, **138**, 5208−5215.
3. A. Alizadeh, A. Burns, R. Lenigk, R. Gettings, J. Ashe, A. Porter, M. McCaul, R. Barrett, D. Diamond, P. White, P. Skeath, M.Tomczak, *Lab Chip* 2018, **18**, 2632−2641.
4. A. A. Stekolshchikova, A. V. Radaev, O. Y. Orlova, K. G. Nikolaev, E. V. Skorb, *ACS Omega* 2019, **4**, 15421−15427.
5. X. Xuan, M. F. Hossain, J. Y Park, *Sci. Rep.* 2016, **6**, 33125.
6. S. T. Mensah, Y. Gonzalez, P. Calvo-Marzal, K. Y. Chumbimuni-Torres, *Anal. Chem.* 2014, **86**, 7269−7273.
7. X. Xuan, J.Y. Park, *Sens. Actuat. B-Chem* 2018, **255**, 1220–1227.
8. D. Zhao, D. Siebold, N.T. Alvarez, V. N. Shanov, W. R. Heineman, *Anal. Chem.* 2017, **89**, 9654−9663.
9. Y. Zhao, X. Yang, P. Pan, J. Liu, Z. Yang, J. Wei, W. Xu, Q. Bao, H. Zhang, Z. Liao, *J. Electron. Mater* 2020, **49**, 6695-6705.
10. W. Gao, H. Y. Y. Nyein, Z. Shahpar, H. M. Fahad, K. Chen, S. Emaminejad, Y. Gao, L. Tai, H. Ota, E. Wu, J. Bullock, Y. Zeng, D. Lien, A. Javey, *ACS Sens*. 2016, **1**, 866−874.
